# Mammalian adaptation risk in HPAI H5N8: a comprehensive model bridging experimental data with mathematical insights

**DOI:** 10.1080/22221751.2024.2339949

**Published:** 2024-04-04

**Authors:** Santosh Chokkakula, Sol Oh, Won-Suk Choi, Chang Il Kim, Ju Hwan Jeong, Beom Kyu Kim, Ji-Hyun Park, Seong Cheol Min, Eung-Gook Kim, Yun Hee Baek, Young Ki Choi, Min-Suk Song

**Affiliations:** a Department of Microbiology, College of Medicine and Medical Research Institute, Chungbuk National University, Cheongju, Republic of Korea; b Department of Biochemistry, College of Medicine and Medical Research Institute, Chungbuk National University, Cheongju, Republic of Korea; c Center for Study of Emerging and Re-emerging Viruses, Korea Virus Research Institute, Institute for Basic Science (IBS), Daejeon, Republic of Korea

**Keywords:** HPAI H5N8, replication, adaptation, RNA load, regression, quantification, risk score

## Abstract

Understanding the mammalian pathogenesis and interspecies transmission of HPAI H5N8 virus hinges on mapping its adaptive markers. We used deep sequencing to track these markers over five passages in murine lung tissue. Subsequently, we evaluated the growth, selection, and RNA load of eight recombinant viruses with mammalian adaptive markers. By leveraging an integrated non-linear regression model, we quantitatively determined the influence of these markers on growth, adaptation, and RNA expression in mammalian hosts. Furthermore, our findings revealed that the interplay of these markers can lead to synergistic, additive, or antagonistic effects when combined. The elucidation distance method then transformed these results into distinct values, facilitating the derivation of a risk score for each marker. *In vivo* tests affirmed the accuracy of scores. As more mutations were incorporated, the overall risk score of virus heightened, and the optimal interplay between markers became essential for risk augmentation. Our study provides a robust model to assess risk from adaptive markers of HPAI H5N8, guiding strategies against future influenza threats.

## Introduction

Influenza A virus (IAV) is a ubiquitous pathogen infecting a wide range of avian species and exhibiting species specificity. Barriers typically constrain the spread of virus between species, yet occasional breaches can lead to severe outbreaks, particularly when avian hosts transmit the virus to humans [1, 2]. Several viral genetic factors, such as the host receptor binding protein (Hemagglutinin, HA) [3, 4], and the polymerase complex [5, 6], are instrumental in the inter-species transmission and adaptation of IAVs. The polymerase complex comprises three proteins: polymerase basic protein 2 (PB2), polymerase basic protein 1 (PB1), and polymerase acidic protein (PA) [7]. Alterations in these proteins significantly influence the cross-species transmission and adaptation of IAV through various host-adaptive viral changes [5, 8–10]. Notably, an amino acid substitution at position 627 of the PB2 gene (E627K) increases polymerase activity, enhancing the replication efficiency of various AIVs in mammalian hosts [11–15]. Additional adaptive mutations in PB2, such as D701N, Q591R/K, T271A, and E158G, along with mutations like P708S in PB1 and T97I, K142E, and I353R in PA, support replication fitness and pathogenicity in mammalian cells and hosts [12, 14, 16–22].

Historically, mathematical and machine learning models have shown immense utility in predicting viral load kinetics, characterizing infection dynamics, modelling host immune responses, and analyzing co-infection kinetics of IAVs [23–25]. These models also estimate vaccine and antiviral therapy effectiveness [26], and predict avian-to-human transmission and other interspecies transmissions by scoring viral protein mutations, including those in the polymerase complex [25, 27]. Incorporating quantitative experimental data, viral kinetic models provide comprehensive insights into influenza virus replication kinetics across various experimental systems [28–30]. These models contribute significantly to our understanding of the dynamic differences across various influenza strains and inform strategies for future pandemic control [31–37].

In this study, we investigated the adaptation of the highly pathogenic avian influenza (HPAI) H5N8 virus in mammalian hosts. Using next-generation sequencing (NGS), we examined viruses from mouse lung samples over five passages. We then generated viruses with specific adaptive markers using reverse genetics (RG) and analyzed their growth, adaptation, and RNA load dynamics. Using regression analysis and the distance method, we derived risk scores for each marker, and validated these scores by assessing recombinant virus pathogenicity *In-vivo*. We also cross-validated our model using another H5N8 virus, RG468, to confirm its accuracy. Our findings provide a clear understanding of adaptive markers in viral pathogenicity. This model is essential for assessing risks from new IAV strains, enhancing surveillance, and guiding preventive strategies.

## Materials and methods

*Cell cultures and viruses*. We cultivated A549 and 293T human cell lines in Dulbecco Modified Eagle Medium, augmented with 10% fetal calf serum and 1% antibiotics. In contrast, MDCK cells were maintained in Eagle Minimum Essential Medium with a 5% FCS addition and 1% antibiotics. Two HPAI H5N8 viruses, specifically A/Mallard duck/Korea/W452/2014 (W452, Accession No. KJ746111) and A/Environment/Korea/W468/2015 (W468, Accession No. KX297870), were utilized in this study.

*Deep sequencing*. To investigate the molecular dynamics of adaptive markers, we utilized lung homogenate samples from each mouse adaptation passage of the mouse-adapted 452 (ma452) and ma468 strains in BALB/c mice (P1-P5). These samples were derived from a prior study [20]. Viral RNA isolation was performed using the RNeasy Minikit (Qiagen) while the conversion of RNA to cDNA was achieved through the MMLV reverse transcriptase kit (Enzynomics), each following the guidelines of manufacturer with minor modifications. PCR amplification and subsequent PCR product purification were conducted using Cosmogenetech DNA purification kit. Next, sequencing libraries were created utilizing the Nextera XT DNA library preparation kit (Illumina). These libraries were then sequenced on the MiSeq platform (Illumina). The raw sequence reads obtained were then processed through several steps: demultiplexing, fast quality check, grooming, and trimming, and filtering. This was performed using the CLC Genomics Workbench 7 software suite (Qiagen, CLC Bio). Finally, all reads were mapped against the W452 and W468 reference sequences. We utilized a quality-based variant detection pipeline to identify any possible mutations.

*Construction of recombinant viruses with adaptive markers*. The recombinant H5N8 viruses, including the wild type (WT) (RG452-WT and RG468-WT) and variants with adaptive markers, were constructed using RG (Supplementary Table 1). This process entailed the amplification of the eight gene segments from the progenitor virus as per the previous studies [38]. Site-direct mutagenesis created a recombinant virus with a mutated polymerase gene. Each polymerase plasmid (PB2, PB1, PA) subjected to PCR (0.1µg per plasmid) with a Phusion enzyme and specific primer for mutagenesis (Supplementary Table 2). Mutated gene segments were cloned after DpnI treatment, verified by sequencing. For transfection, 1µg of each mutated or wild-type polymerase plasmid and 1µg of remaining gene plasmids were introduced using Lipofectamine in Opti-MEM medium. Co-culture with a 3:1 ratio of 293T and MDCK cells occurred at 37°C with 5% CO_2_. After 18 h, supernatants were replaced, and 48 h post-transfection, TPCK trypsin was added. Supernatant was propagated in SPF embryonated chicken eggs, stored at −80°C, and titres were determined by TCID_50_ assays in MDCK cells, expressed in log_10_.

*Development of a model*. We examined the growth kinetics, competitive selection intensity, and RNA load of both individual adaptive markers and their combinations. The development and validation of our model involved the following experimental and mathematical systems.

### Growth kinetics and regression analysis

In the analysis of growth kinetics, both recombinant RG452-WT and variant viruses were used to infect monolayers of A549 cells with a multiplicity of infection (MOI) of 0.01. After a one-hour incubation at 37°C, the supernatants were replaced with an infection medium that did not contain TPCK-trypsin. Cell culture supernatants were harvested at 12, 24, 36, 48, and 60 days’ post infection (dpi). Viral titres were determined on MDCK cells and expressed as Log_10_TCID_50_/mL. Growth speed (GS) of the virus were evaluated using regression analysis.

The growth pattern of the influenza virus, unlike that of other viruses, follows an S-shaped trajectory, prompting the use of a logistic model using the “growth rate” package in R. Viral titre was measured at 12-hour intervals from 0 to 60 h. The following model was assigned for the expression of growth kinetics:

$$Y=\frac{y_{0}.\;K}{y_{0}+(K-y_{0}).\;e^{-m.\mathrm{time}}}$$

In this model, “*K*” denotes the maximum titre value, “*y*_0_” signifies the minimum value, “time” is the end time, and “*m*” is a parameter derived through regression analysis. The stay period for growth is 1.8 times. This value was considered as the “stay” time to better reflect the growth pattern of virus, transforming the equation for replication kinetics to:

$$Y=\frac{y_{0}.\;K}{y_{0}+(K-y_{0}).\;e^{-m.(\mathrm{time}-\mathrm{stay})}}$$

Here, “stay” represents the stay time, with all other variables remaining as previously defined.

This equation only depicts the GS during the influenza virus growth phase, excluding the stay phase. Therefore, it was necessary to determine a new growth rate for the influenza virus. Thus, the average GS of the influenza virus was obtained using the following model, which employed estimated values derived from regression analysis of the influenza virus:

$$Y_{n}=\;(s+1{)}^{n-1}.\;y_{0}$$

In this equation, “*n*” represents the maximum time and “*s*” is the average GS of the influenza virus.

### Competitive selection kinetics and its regression analysis

A549 cells were transfected with recombinant RG452-WT and variant viruses at three different ratios (1:100, 1: 1,000, and 1: 10,000). Following transfection, supernatants were replaced with an infection medium. After three days of incubation, the supernatants were collected and used to infect A549 cells for five consecutive passages. The viral stocks were stored at −80°C for subsequent analyses.

To estimate the cellular competitive selection rate of the viruses containing adaptive markers, we passaged the viruses and employed index sequencing with NGS for the 0^th^, 1^st^, 3^rd^, and 5^th^ passages. We set a threshold value of 2, performing random sampling 100 times, and thus developed a model with a minimum value of 2 and a maximum value of 100 for the logarithmic growth curve. The equation used for this model is:

$$Y=\;-e^{\left(-(\mathrm{slope}.(\;\mathrm{passage})-\log(98))\right)}+100$$

In this model, the “slope” represents the rate at which influenza virus particles carrying the mutation adapt to the cells. The passage number at which the virus adapts and acquires mutation stability is denoted as “stay.” Considering this “stay” phase, we created a curve using a model with a threshold value of 2, where the mutation proportion is close to 100%. The model representing the “stay” phase for graphical representation is:

$$Y=\;-e^{\left(-(\mathrm{slope}.(\;\mathrm{passage}-\max(\mathrm{stay}))-\log(98))\right)}+100$$

To assess the competitive selection intensity of RG452 variant viruses against RG452-WT, values were consolidated into a singular metric by determining the area under the curve (AUC) values. These AUC values subsequently illustrated the competitive selection rate of the recombinant variants equipped with adaptive markers in comparison to RG452-WT.

### RNA load kinetics and regression analysis

A549 cells were infected with RG452-WT and variant viruses, each at MOI of 0.1. Four hours’ post-infection, infected cells were collected and their total RNA was extracted using a Qiagen RNeasy Minikit. cDNA was synthesized from the NP viral RNA segment with gene-specific primers (Supplementary Table 3). Quantitative real-time PCR was then conducted on a LightCycler® 96 system (Roche), in accordance with previously described methods and conditions [39].

A549 cells were infected with RG452-WT and variant viruses, each at a MOI of 0.1. Four hours’ post-infection, infected cells were collected and their total RNA was extracted using a Qiagen RNeasy Minikit. For cDNA synthesis of vRNA, cRNA, and mRNA, mix 10 ul of RNA, 1 ul (10 pmol) of Revese Transcription primer, and 4 ul of distilled water heat at 65°C for 10 min, immediately cool on ice for 5 min, and then add 2 μl dNTP mixture (10 mM each), 1 μl Superscript III enzyme, 2 ul DDT (0.1M) were mixed and heated at 60°C for 1 h. To inhibit the enzyme, it was incubated at 70°C for 10 min. Quantitative real-time PCR was then conducted on a LightCycler® 96 system (Roche) with the assistance of Superscript III Reverse Transcriptase (Life Technologies) and FastStart SYBR Green Master (Sigma-Aldrich), in accordance with previously described methods and conditions [39]. The 3 ul of 10-fold diluted cDNA was added to the qPCR reaction mixture containing 10 μl SYBR GreenER qPCR SuperMix, 2 μl forward primer (5 μM), 2 μl reverse primer (5 μM), 3 μl double distilled water. Cycling conditions for qPCR were 95°C for 10 min, followed by 40 cycles of 95°C for 15 s and 60°C for 1 min. A standard curve was generated using 10-fold serial dilutions (10^9^, 10^8^, 10^7^, 10^6^, 10^5^, 10^4^, and 10^3^ copies/μl) of synthetic viral RNA standards.

After relative quantitation of the RNA, we then conducted a regression analysis, incorporating metrics from GS and competitive selection rate (CSR) as dependent variables and the RNA fold change values as independent variables to achieve the weight of each viral RNA type.

The weights assigned to each RNA type were determined by the arithmetic mean of the values gleaned from the regression analysis. The model is as follows:

log (replication kinetics)=weight1∗RNA fold change

log (competition assay)=weight2∗RNA fold change

In this case, weight1 and weight2 are parameters obtained from the regression analyses of the replication kinetics and competition kinetics against RNA fold change, respectively. Following the attainment of the weights for cRNA, vRNA, and mRNA via regression analysis, the sum of these ratios was utilized to create a comprehensive RNA load value.

### Assessment of quantitative risk scores

The parametric values gleaned from the regression analyses – GS, CSR, and RNA load value – were all scaled to one. These scaled values were then subjected to a calculation of Euclidean distance, generating quantitative risk scores for all adaptive markers, as per the following model:

$$d=\;\sqrt{{\left(x_{2}-x_{1}\right)}^{2}+{\left(y_{2}-y_{1}\right)}^{2}+{\left(z_{2}-z_{1}\right)}^{2}}$$

In this equation, “*d*” represents the Euclidean distance. The ordered pairs (x_1_, y_1,_ z_1_) and (x_2_, y_2_, z_2_) denote the initial and final coordinate points, respectively. This model essentially offers a straightforward way to quantify the “distance” or difference between the values of adaptive markers, enabling a more systematic and mathematically rigorous assessment of their respective risk levels.

*Cross-validation of the model*. We cross-validated our model using another H5N8 virus, RG468, to confirm its ability to accurately predict the quantitative risk scores (QRS) of all adaptive markers for the influenza A virus. Similar to RG452, we extracted all metrics such as GS, CSR, RNA, risk score for RG468. Overall, cross-validation with six recombinant RG468 viruses with the adaptive markers and the statistical parameters affirm the reliability and sensitivity of the model in predicting the quantitative scores of all adaptive markers for influenza A virus.

### In vivo model validation: sensitivity and specificity analysis

#### Animal subjects

The study used 6–7-week-old female BALB/c mice procured from the Jackson Laboratory (Bar Harbor, Maine, USA) to validate the model *in vivo*. We split the mice into eight experimental groups, with five mice per group (*N* = 5), to analyze weight change and survival. Additionally, for systemic infection analysis of the recombinant RG452 viruses, we formed eight more groups of three mice each (*N* = 3).

#### Survival, weight change, systemic infection analysis of the RG452 viruses

Mice were intranasal infected with five varying viral titres of eight distinct recombinant RG452 virus, ranging from 1 to 6 log_10_TCID_50_/mL, and monitored over for 14 days for weight change, mortality, and other signs of morbidity. In a separate experiment, mice were infected with a 4 log_10_TCID_50_/mL virus dose, and the systemic spread of the virus was examined in various organs, including the lungs, brain, heart, spleen, and kidneys, which were collected at 5 dpi for viral titre estimation.

#### Performance evaluation of the risk scores of each recombinant RG452 virus containing the single and multiple adaptive markers

We compared *In vitro* QRS with *In vivo* data on survival rates, weight changes, and systemic viral spread to validate the model. Receiver operating characteristic (ROC) curves were generated, plotting sensitivity against the relative false-positive rate (1-specificity) [40]. The AUC measures the predictive accuracy of each adaptive marker. A ROC curve passing through the upper left corner, corresponding to an AUC of 1, indicates an ideal quantitative trait with 100% sensitivity and 100% specificity.

### Statistical analysis

We utilized the rstatix package in R to obtain the ANOVA summary statistical information across various study objects. The statistical significance between the wild type and other adaptation markers was executed using a *t*-test of the ggsignif package in R. The survminer package in R was utilized for Kaplan–Meier analysis to assess overall survival across the study groups. Significance in the difference of overall survival was determined using the log-rank test. For all the study groups, statistical significance when compared to RG452-WT is denoted by “ns” for non-significant, *p < 0.05, **p < 0.01, and ***p < 0.001.

## Results

### Dynamics of mammalian adaptive markers during sequential passage of H5N8 viruses in mice

**Figure 1.**
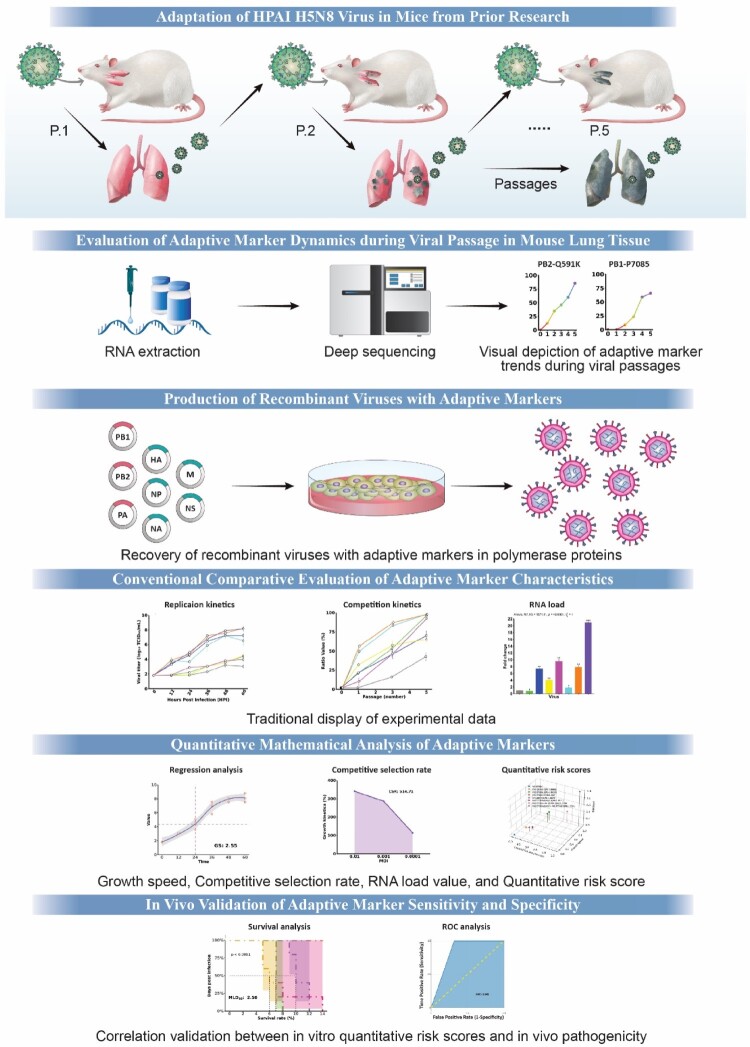
Study overview. This research delves into mice adaptation to identify adaptive markers using NGS. Utilizing reverse genetics, we generated viruses with various adaptive markers to conduct three experiments: replication dynamics, adaptation analysis, and RNA quantification. A nonlinear regression model was then applied to derive metrics, which were used to compute individual QRS for each marker. *In vivo* experiments subsequently validated these scores for sensitivity and specificity. GS-growth speed, QRS-quantitative risk scores.

**Figure 2.**
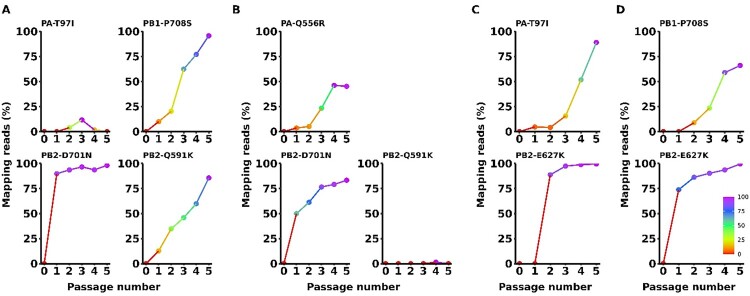
Mapping intensity of adaptive markers in ma452 and ma468 viruses through five sequential passages. The figures display an escalating mapping intensity for specific adaptive markers in viruses: (A) ma452-G1, (B) ma452-G3, (C) ma468-G1, and (D) ma468-G2. The deep-sequencing was performed using sequentially passaged mice lung samples in previous study. Each of these viruses embodies unique adaptive markers situated within their viral polymerase genes. ma denotes mouse-adapted.

Figure 1 offers a comprehensive schematic representation of the study. We previously conducted mouse adaptation studies using two HPAI H5N8 viruses, W452 and W468 [20]. These viruses were serially passage through four mouse groups for five consecutive passages (P1–P5). In this current study, lung sample homogenates from two out of the four mouse groups for each adaptation passage were subjected to NGS to confirm the mutations leading to amino acid substitutions. In the W452-WT infected mouse groups, the PB2_D701N_ substitution, a recognized adaptive mutation [41], was observed with a frequency of 50-90% after just one passage (Figure 2(A,B)). Alongside PB2_D701N_, the PB2_Q591K_ mutation, another recognized mammalian adaptive mutation [17], was detected, although its frequency increased more gradually, only becoming predominant (over 50% of the population) after the fourth passage in one of the mice (Figure 2(A,B)). PB1_P708S_ mutation was detected in both virus passage groups and also gradually increased, resulting in 60-95% of the population after five passages (Figure 2(A,D)). The PB2_E627K_ substitution, a well-documented mammalian adaptive marker [2], was exclusively detected in the W468-WT infected mouse groups. It became predominant (over 85% of the population) within just two passages, ultimately representing nearly 100% of the viral population (Figure 2(C,D)). Though the PA_T97I_ substitution was detected in both virus passage groups, it only dominated (over 80%) when combined with PB2_E627K_ (Figure 2(A,C)). Lastly, the PA_Q556R_ mutation was recently found in one of the W452-WT infected mice but never represented more than 50% of the viral population (Figure 2(B)). These results demonstrate that each adaptive marker correlates with differential viral dynamics, influencing the intensity, frequency, and viral state within the host.

### Growth speed values facilitate quantitative analysis of distinct replication kinetics in recombinant viruses with various mammalian adaptive markers

**Figure 3.**
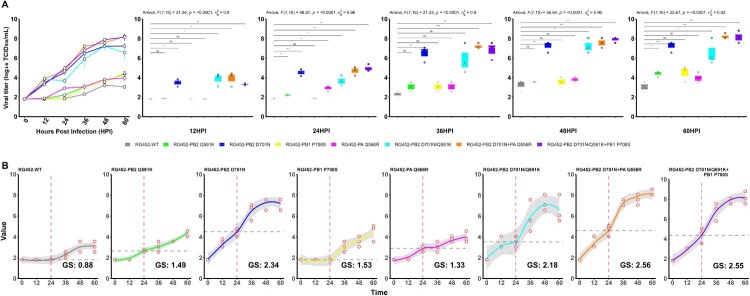
In vitro growth dynamics and speed of recombinant viruses with single and combined adaptive markers. (A) This panel displays the traditional *In vitro* growth kinetics of eight recombinant viruses, each with distinct single or combined markers, produced via reverse genetics. This kinetics are plotted over various time intervals: 12HPI, 24 HPI, 36 HPI, 48 HPI, and 60 HPI. Statistical relevance when compared to RG452-WT is signified with asterisks (*p < 0.05, **p < 0.01, ***p < 0.001, ****p < 0.0001); “ns” represents non-significance. HPI is an abbreviation for Hours Post Infection. (B) This segment showcases the logistic nonlinear regression analysis applied to recombinant RG452 viruses with adaptive markers, using time vs. titre data to derive GS values. This analysis evaluates the adaptive capacity of various RG452 markers, listing them alongside their respective titres. GS denotes growth speed.

Eight recombinant viruses, each embodying distinct adaptive markers, were produced using the RG technique with the background of A/EM/W452/Korea/14 (H5N8, W452) virus (Supplementary Table 1). To analyze the replication capacity of these RG452 viruses, including WT, that harbour either individual or multiple adaptive markers, they were introduced to A549 cells at a MOI of 0.01. The RG452 viruses endowed with individual mutations, such as PB2_Q591K_, PB1_P708S_, and PA_Q556R_, displayed replication patterns either equivalent to or marginally superior to RG452-WT. The RG452-PB2_D701N_ virus, however, exhibited the most pronounced replication advantage in comparison to both RG452-WT and other single mutants, at all-time points evaluated (Figure 3(A,B)). The highest replicative competence was displayed by the triple mutant RG452-PB2_Q591K/D701N _+ PB1_P708S_ and the double mutant RG452-PB2_D701N _+ PA_Q556R_, followed by the double mutant RG452-PB2_Q591K/D701N_ (Figure 3(A)). Although the combination of multiple markers in RG452 viruses generally enhanced replication compared to single mutants, the RG452 virus with the double mutations RG452-PB2_Q591K/D701N_ interestingly exhibited a similar to slightly lower proliferation efficiency relative to the single mutant RG452-PB2_D701N_ (Figure 3(A,B)). Our observations highlight the crucial role of the PB2_D701N_ mutation in boosting viral growth potential, typically synergizing effectively with other mutations. Nonetheless, we noted potential antagonistic replication when the PB2_D701N_ mutation was combined with the PB2_Q591K_ mutation. This antagonism was mitigated upon inclusion of the PB1_P708S_ mutation, as observed in the triple mutant RG452-PB2_Q591K/D701N _+ PB1_P708S_ (Figure 3(A,B)).

The replication kinetics and growth potential of all these viruses can be succinctly interpreted through straightforward graphical representations. Acknowledging the advantages of quantitative comparison, we assessed replication capacity using non-linear regression analysis. Our analyses yielded R^2^ values ranging from 0.85 to 0.98 (Supplementary Table 4), suggesting the curves were well-fitted and displayed superior replication kinetics for all the viruses. We then computed the average growth rate spanning the duration from when an uptick in viral amount was first observed to the point where a peak viral amount was achieved. This allowed us to estimate the average hourly virus production capacity. Among the single mutants, the RG452-PB2_D701N_ virus displayed the most accelerated growth speed (GS = 2.34). This was followed by the RG452-PB1_P708S_ (GS = 1.53), PB2_Q591K_ (GS = 1.49), and PA_Q556R_ (GS = 1.33) viruses (Figure 3(C)). Considering the multiple mutants, both RG452-PB2_D701N _+ PA_Q556R_ (GS = 2.56) and PB2_Q591K/D701N _+ PB1_P708S_ (GS = 2.55) viruses exhibited greater growth speed, followed by the double mutant RG452-PB2_Q591K/D701N_ virus (GS = 2.18) (Figure 3(C)). Interestingly, the GS of the double mutant RG452-PB2_Q591K/D701N_ virus was lower than its single mutant counterpart, RG452-PB2_D701N_, aligning with the viral growth kinetics observed earlier (Figure 3(C)). We noted that each single adaptive marker conferred unique quantitative characteristics to GS, and the introduction of additional markers influenced the speed of replication. The PA_Q556R_ single mutant exhibited a lower GS than other single mutants. However, its GS was significantly boosted when combined with the PB2_D701N_ mutation (RG452-PB2_D701N _+ PA_Q556R_), reaching one of the highest GS values among the RG452 viruses (Figure 3(C)).

### Quantitative comparison utilizing competitive selection rate accurately mirrors the relative selectivity of mammalian adaptive markers

**Figure 4.**
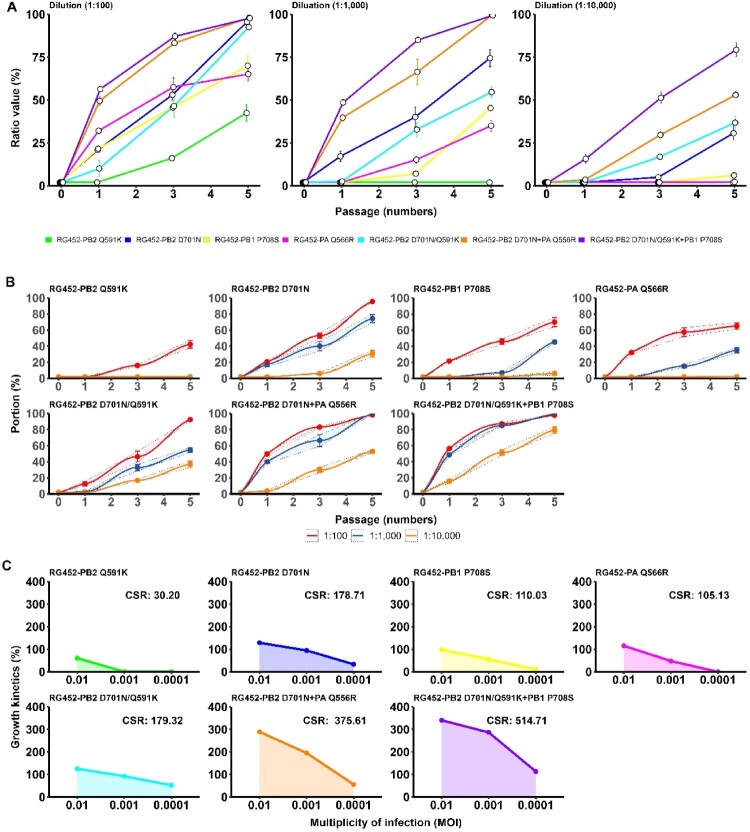
Competitive selection and kinetics of adaptive markers in RG452 viruses. (A) Competitive Selection Dynamics: The panel displays *In vitro* competitive selection intensities of individual and combined adaptive markers for recombinant RG452 viruses. Selection intensities of these markers are depicted when diluted at ratios of 1:100, 1:1000, and 1:10000 against the wild-type RG virus, revealing the competitive prowess of each adaptive marker. (B) CSK: Here, the nonlinear regression analysis evaluates the selection kinetics of various RG452 adaptive markers, including RG452-WT, RG452-PB2_Q591K_, RG452-PB2_D701N_, RG452-PB1_P708S_, and others. Analysis was carried out at three concentration ratios: 1:100, 1:1000, and 1:10000, utilizing the index sequence value as the criterion. (C) AUC Analysis: This segment showcases the AUC plots derived from adaptation values across different MOIs. The resultant AUC values corresponding to each adaptive marker are enumerated. AUC refers to the area under the curve.

To explore further into the influence of individual and combined mammalian adaptive markers present in the HPAI H5N8 virus on the host specificity in humans, we conducted competitive selection kinetics (CSK) studies. For this, we exposed A549 human lung adenocarcinoma cells to mixtures of RG452-WT and mutant viruses in dilutions of 1:100, 1: 1,000, and 1: 10,000 and performed five sequential passages. The NGS was employed to evaluate the proportions of mutant viruses in the supernatants of the 1st, 3rd, and 5th passages. The results demonstrated that among single mutants, RG452-PB2_Q591K_ had the least competitive edge against RG452-WT, as evident from the competitive selection observable only at the least dilution concentration of 1:100. This was followed by RG452-PB2_Q556R_, and RG452-PB1_P708S_, which showed competitive selection until a 1: 1,000 dilutions (Figure 4(A)). Remarkably, the single mutant RG452-PB2_D701N_ revealed clear competitive selection up to the highest dilution concentration of 1: 10,000 (Figure 4(A)). Examining multiple mutants, it was observed that they all exhibited clear competitive selection until a 1: 10,000 dilutions. However, the triple mutant RG452-PB2_Q591K/D701N _+ PB1_P708S_ outperformed others by displaying the most pronounced competitive selection across all dilution concentrations (Figure 4(A)). This evidence suggests that the mutation PB2_D701N_ enhances host selectivity at lower viral concentrations more than other single mutations, and its effect is further amplified when combined with additional mutations.

To quantify the effect of CSK on the mammalian adaptive markers, we employed a two-step quantitative conversion. We initially conducted a nonlinear regression analysis (Figure 4(B)) to calculate the CSK value of all mutants, considering the viral concentration at passages for each mutant. Then, we computed the competitive selection rate (CSR) of the mutants from the AUC, using the CSK values at dilutions of 1:100, 1: 1,000, and 1: 10,000. The R² values (0.8∼0.9) obtained from the regression analysis were within acceptable limits, confirming the suitability of the model. When examining the single mutants, all displayed CSK values ranging between 60.4 and 128.6 (*R*² = 0.88–0.96) at the lowest dilution point (1:100) (Supplementary Table 5). However, most of them, with the exception of the single RG452-PB2_D701N_ mutant, experienced a rapid decline in CSK values at higher dilutions (1: 1,000 and 1: 10,000) (Figure 4(B) and Supplementary Table 5). On the other hand, the multiple mutants presented higher CSK values ranging from 125.4 to 340.4 (*R*² = 0.85–0.99) at the lowest dilution and consistently maintained higher CSK values at increased dilutions compared to the single mutants (Figure 4(B) and Supplementary Table 5). Nonetheless, the double RG452-PB2_Q591K/D701N_ mutant recorded values akin to the single RG452-PB2_D701N_.

The CSR value, obtained by calculating the AUC from three CSK values, effectively illustrates the relative selectivity of the mutants and further validates the effectiveness of the model (Figure 4(C)). Among the single mutants, RG452-PB2_D701N_ demonstrated the highest CSR value (CSR = 178.71), followed by RG452-PB1_P708S_ (CSR = 110.03), RG452-PB2_Q556R_ (CSR = 105.13), and RG452-PB2_Q591K_ (CSR = 30.20) mutants (Figure 4(C)). Observations of combinatory mutations show various effects. An antagonistic effect was observed in the double RG452-PB2_Q591K/D701N_ mutant (CSR = 179.32). Additive effects were identified in the RG452-PB2_D701N _+ PA_Q556R_ mutant (CSR = 375.61), and synergistic effects were present in the triple RG452-PB2_Q591K/D701N _+ PB1_P708S_ mutant (CSR = 514.71).

### The RNA weight model enhances the RNA scoring reliability of m, c, and vRNA through the influence of mammalian adaptive markers

**Figure 5.**
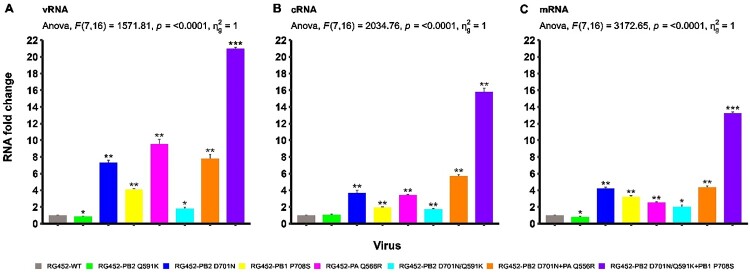
RNA expression levels of adaptive markers in RG452 virus. (A) vRNA Expression Dynamics: This panel depicts the fold change intensity of vRNA for each single and combined adaptive marker in the RG452 virus. (B) cRNA Expression Dynamics: Similarly, this segment displays the cRNA fold change intensity associated with each adaptive marker. (C) mRNA Expression Dynamics: This section presents the mRNA fold change intensity measurements for each adaptive marker. For all panels, statistical significance when compared to RG452-WT is denoted by: “ns” for non-significant, *p < 0.05, **p < 0.01, and ***p < 0.001.

Given the significance of viral RNA expression levels in influencing viral kinetic properties, we analyzed the expression of mRNA, cRNA, and vRNA across all recombinant RG452 viruses, including the WT. RG452 viruses with mammalian adaptive mutations displayed a higher RNA expression profile than the RG452-WT, whereas the RG452-PB2_Q591K_ showed a comparable RNA profile relative to the RG452-WT. In parallel to GS and CSR, RG452-PB2_D701N_ demonstrated the highest expression levels of cRNA and mRNA among single mutants, even though its vRNA profile was lower than that of RG452-PA_Q556R_. The single PA_Q556R_ mutation was found to have a more pronounced effect on the RNA expression pattern, compared to its impact on GS and CSR. The negative synergy between the PB2_Q591K_ and PB2_D701N_ mutations was especially evident in the viral RNA profile, a drawback that was significantly offset by the incorporation of the PB1_P708S_ mutation (RG452-PB2_D701N/Q591K _+ PB1_P708S_) (Figure 5).

We first validated the weight of each viral RNA to accurately quantify the intra-cellular expression patterns of m, c, and vRNA, highlighting the unique influence of each RNA type on viral attributes. Using RNA fold change values from each virus and GS in our regression analysis, we obtained an R² value under 1 and achieved statistical significance (*p* < 0.002) (Table 1). This indicates a dependable expression profile across the three RNA types. The robustness of our model was further underscored by regression analyses of RNA fold change values associated with each virus and CSR, which consistently yielded significant R² values and *p*-values (*p* < 0.001) (Table 1). Notably, the regression values for RNA-GS and RNA-CSR in each RNA type were nearly identical, emphasizing the accuracy of RNA weight estimation through our regression approach. We then defined the average quantitative RNA-GS and RNA-CSR regression values as RNA weight. Here, cRNA registered the highest weight, trailed by mRNA and vRNA, with respective weights of 0.38, 0.34, and 0.28 (Table 1).

**Table 1.** RNA weight model based on three RNA types and RNA load values for single and combination adaptive markers of RG452.

RNA type	RNA^a^ weight (average)	RNA-GS regression		RNA-CSR regression		RNA load value (RNA fold change)						
		Estimated RNA-GS	*R*-squared	Estimated RNA/CSR	*R*-squared	RG452-PB2_R591K_	RG452-PB2_D701N_	RG452-PB1_P708S_	RG452-PA_Q556R_	RG452-PB2_D701N/R591K_	RG452-PB2_D701N _+ PA_Q556R_	RG452-PB2-_D701N/Q591K_ + PB1_P708S_
mRNA	0.34	0.34	0.5573	0.34	0.5819	0.24^c^	1.2	0.83	0.72	0.54	1.21	3.57
						(0.86)^d^	(4.32)	(2.99)	(2.57)	(1.95)	(4.35)	(12.83)
cRNA	0.38	0.37	0.7834	0.38	0.8642	0.41	1.38	0.73	1.32	0.68	2.18	5.95
						(1.07)	(3.62)	(1.92)	(3.47)	(1.79)	(5.73)	(15.63)
vRNA	0.28	0.29	0.7768	0.28	0.7294	0.3	2.51	1.39	3.26	0.63	2.66	7.44
						(0.87)	(7.34)	(4.07)	(9.56)	(1.85)	(7.81)	(21.82)
Total RNA	1	1	*P* < 0.002^b^	1	*P* < 0.001	0.94^e^	5.08	2.95	5.3	1.86	6.05	16.96

GS; Growth Speed, CSR; competitive selection rate.

^a^ The RNA weights were calculated using the average values from the RNA-GS and RNA-CSR regressions for each RNA type.

^b^ *P*-values indicate the statistical significance of the RNA-GS and RNA-CSR regressions across all RNA types.

^c^ Values indicate the RNA load from each RNA type for each recombinant virus.

^d^ Values depict the fold change in RNA levels for each type of the specified recombinant virus relative to the wild type RG452 for each RNA type.

^e^ Values show the aggregated RNA load from all RNA types for each recombinant virus.

An RNA load value was ascertained by evaluating the mean values of mRNA, cRNA, and vRNA for wild-type and mutant viruses. Among the single mutants, RG452-PB2_D701N_ and RG452-PA_Q556R_ achieved the highest RNA load values (Table 1). Although the combined PB2_D701N_ and PA_Q556R_ mutations led to an improved RNA load value, the enhancement was only modest (Table 1). Conversely, the RNA load value of RG452-PB2_D701N/Q591K_ notably diminished when the PB2_Q591K_ mutation was incorporated. This decline was significantly counteracted with the combination of triple mutations (RG452-PB2_D701N/Q591K _+ PB1_P708S_), resulting in substantial improvement among both single and multiple mutants (Table 1).

### The quantitative risk scores can effectively represent the magnitude of mammalian adaptive marker’s effect on the viral properties

**Figure 6.**
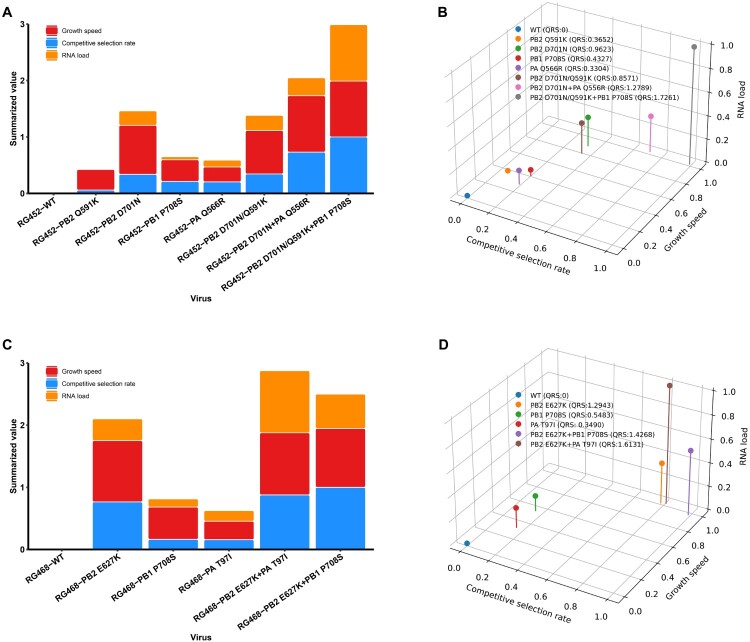
Scaled scores and tri dimensional representation of RG452 and RG468. (A) The scaled scores of replication, competition, and RNA kinetics values of the single and combination adaptive markers of the RG452. (B) The tri dimensional representation of the single and combinational adaptive markers associated with replication, competition, and RNA kinetics of the RG452 yielded the QRS of each adaptive markers of the RG452. (C) The normalized scaled scores of replication, competition, and RNA kinetics of the single and combinational adaptive markers of the RG458 (D) The three-dimensional depiction of the single and combinational adaptive markers linked to replication, competition, and RNA kinetics within of RG468 generated the QRS of each adaptive marker of the RG468. QRS refers to the quantitative risk scores.

Finally, quantitative risk scores (QRS) were derived using regression parameters associated with GS, CSR, and RNA load values. Each of these three parameters was scaled to a value of 1, allowing for a maximum cumulative value of 3. Among the single mutants, RG452-PB2_D701N_ scored the highest, while RG452-PB2_D701N _+ PA_Q556R_ led among the double mutants, and the triple mutant RG452-PB2_Q591K/D701N _+ PB1_P708S_ scored the highest value across all other mutants (Figure 6(A) and Supplementary Table 6). The model was then evaluated using a three-dimensional Euclidean distance calculation, generating QRS for all adaptive markers (Figure 6(B)). PB2_D701N_, a dominant mutation commonly found in avian influenza viruses isolated from mammalian hosts, displayed the highest QRS (0.9623) compared to other single mutations. The triple mutant RG452-PB2_Q591K/D701N _+ PB1_P708S_ ranked highest (QRS = 1.7261) among all single and multiple mutants (Figure 6(B)). While PB2_D701N_ mutation alone conferred the highest QRS, it exhibited an antagonistic effect when combined with PB2_Q591K_ (QRS = 0.8571). Conversely, the same PB2_D701N_ mutation was linked to an additive effect when co-occurring with PA_Q556R_ (QRS = 1.2789). The single mutations PB2_D701N_, PB2_Q591K_ (QRS = 0.3652), and PB1_P708S_ (QRS = 0.4327) demonstrated distinct QRS, and in combination, the triple mutants displayed an additive effect, achieving the highest overall QRS (Figure 6(B)). The evaluation and interpretation of this model revealed that each adaptive marker can be characterized by a specific risk score, and the single digital values accurately represent the experimentally characterized viral properties. Generally, the emergence of additional mutations correspondingly increases the QRS of virus, thereby enhancing the ability to predict its adaptive potential.

### Cross-validation of quantitative risk scores with RG468 virus and its adaptive markers confirms perfect accuracy

The QRS model, developed and evaluated using RG452-WT and its mutants, were further cross-validated with wild-type virus RG468 and associated adaptive markers PB2_E627K_, PB1_P708S_, PA_T97I_, PB2_E627K _+ PB1_P708S_, and PB2E_627K _+ PA_T97I_. The consistency observed in the metrics generated by this model substantially contributes to its accuracy, underscoring its importance in risk assessment. In alignment with predictions from the developed model, all mutant viruses demonstrated a higher QRS compared to the RG468-WT virus (Figure 6(B,C) and Supplementary Table 7). Particularly, PB2_E627K_, known as a major mammalian adaptive marker, conferred a higher QRS (1.2943) among single mutants. The double mutant RG468-PB2_E627K _+ PA_T97I_ exhibited higher QRS (1.6131) compared to RG468-PB2_E627K _+ PB1_P708S_ (QRS = 1.4268). In congruence with the adaptive markers observed with RG452, additive QRSs were also noted with the combination of mammalian adaptive markers, reflecting patterns observed in *in vitro* viral characteristics.

### In vitro viral properties correlate with in vivo phenotypes providing adequate sensitivity and specificity yields for the quantitative risk scores of the various mammalian adaptive markers

**Figure 7.**
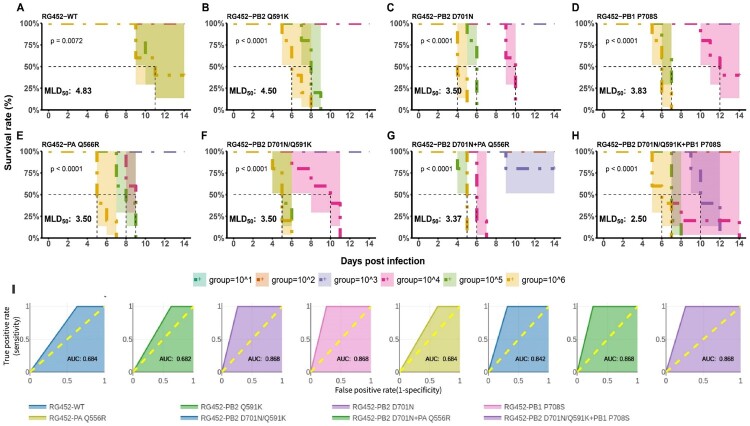
Survival analysis and model evaluation for adaptive markers in BALB/c mice. (A–H) Survival Metrics of RG452 Adaptations: Each panel showcases the survival rates of BALB/c mice after inoculation with varying dilutions (from 10^6^ to 10^1^) of the RG452 recombinant viruses, each carrying distinct adaptive markers: (A) RG452-WT, (B) RG452-PB2_Q591K_, (C) RG452-PB2_D701N_, (D) RG452-PB1_P708S_, (E) RG452-PA_Q556R_, (F) RG452-PB2_D701N/Q591K_, (G) RG452-PB2_D701N_ + PA_Q556R_, (H) RG452-PB2-D701N/Q591K + PB1_P708S_. Survival patterns were monitored for 14 dpi. The Kaplan–Meier survival plots provide insights into the survival rates for each adaptive marker group. (I) ROC Curve Analysis: The graph contrasts the false positive rate (x-axis) with the true positive rate (y-axis) to evaluate the performance of the model built on weight, multi-organ titre, and survival metrics of RG452. The AUC values for each RG virus variant, listed sequentially in the figure, shed light on the sensitivity and specificity of the associated risk scores.

The *In vitro* viral properties associated with mammalian adaptive markers were precisely evaluated to validate their correlation with the *In vivo* phenotypes of the viruses. The 50% mouse lethal dose (MLD_50_) was determined using viral concentrations ranging from 1 to 6 log_10_TCID_50_/mL. Survival and weight loss of infected mice were monitored over a 14-day period, with humane euthanization if their body weight fell below 80% of the initial value. Infection with the high doses (6 and 5 log_10_TCID_50_/mL) of RG452-WT virus led to peak weight loss from 8-10 dpi, with a survival rate of 40% (Figure 7(A) and Supplementary Figure S1(A)). All single mutants exhibited enhanced lethality compared to RG452-WT (Figure 7(A–E)). Among them, RG452-PB2_Q591K_ was the least lethal (Figure 7(B)), while RG452-PB1_P708S_ displayed greater lethality (Figure 7(D)). RG452-PB2_D701N_ and RG452-PA_Q556R_ were the most lethal among single mutants (Figure 7(C,E)). Consistent with *In vitro* findings, the RG452-PB2_Q591K/D701N_ double mutant presented similar weight loss and lethality to the single RG452-PB2_D701N_ mutant (Figure 7(C,F)). Conversely, another double mutant, RG452-PB2_D701N _+ PA_Q556R_, manifested increased lethality and weight loss (Figure 7(G) and Supplementary Figure S1(G)). The triple mutant RG452-PB2_Q591K/D701N _+ PB1_P708S_ demonstrated the most pronounced weight loss and heightened lethality at doses of 3, 4, 5, and 6 log_10_TCID_50_/mL (Figure 7(H) and Supplementary S1(H)). Systematic analysis of the viral doses and their effects on weight and survival led to the calculation of MLD_50_ values as follows: 4.83, 4.5, 3.5, 3.83, 3.5, 3.5, 3.37, and 2.5 for RG452-WT, -PB2_Q591K_, -PB2_D701N_, -PB1_P708S_, -PA_Q556R_, -PB2_Q591K/D701N_, -PB2_D701N _+ PA_Q556R_, and -PB2_Q591K/D701N _+ PB1_P708S_, respectively.

To further assess systemic viral spread, mice were infected with 4 log_10_TCID_50_/mL and their organs (lungs, heart, brain, spleen, and kidney) were harvested and titrated at 5 dpi. While no systemic infection was observed in RG452-WT and -PB2_Q591K_-infected mice, those infected with RG452-PA_Q556R_ and -PB1_P708S_ showed moderate spread and low organ titres (Supplementary Figure S2). Conversely, RG452-PB2_D701N_ had the highest viral titre in multiple organs among the single mutants. Multiple mutants, namely RG452-PB2_Q591K/D701N _+ PB1_P708S_, -PB2_D701N _+ PA_Q556R_, and -PB2_Q591K/D701N_, demonstrated escalating systemic infections in that order (Supplementary Figure S2).

*In vivo* assessments, which included lethality, weight loss, and systemic infection in vital organs, were used to evaluate the performance of the obtained QRS for RG452-WT and its mutant strains. Utilizing a ROC analysis, which plots sensitivity against 1-specificity, most viral strains exhibited ROC curves near top-left corner of the graph (Figure 7(I)). This location signifies accurate risk discernment for each virus type. Quantitatively, the AUC values for single and multiple mutations ranged from 0.682 to 0.868 (Figure 7(I)), underscoring the efficacy of our risk model in relation to *In vivo* observations. Moreover, sensitivity and specificity of the model crucial for prediction model evaluation, were consistently reliable across the mutant strains, enhancing the credibility of our model in assessing risks associated with mammalian adaptive markers.

## Discussion

IAV, particularly the HPAI H5N8 subtype, have attracted significant attention due to their potential for cross-species transmission and adaptation. Central to these phenomena are adaptive mutations within their polymerase complex, which consists of essential proteins such as PB2, PB1, and PA. Prior research underscores the pivotal roles of these proteins in cross-species dynamics of IAV [5, 6, 7, 20, 42, 43]. Building on this foundational knowledge, our study focuses on mutations, including the well-documented PB2 mutations like E627K and D701N [15], known to have a significant influence on mammalian adaptation. Through our studies, we’ve observed the broad spectrum of adaptive mutations within the HPAI H5N8 virus. In particular, the mutations PB2_D701N_ and PB2_Q591K_ in infected mouse groups showcase the differential influence of these mutations on host viral dynamics. We’ve also identified an upward trend in PB2_E627K_ and PB2_D701N_ mutations, indicating their distinct evolutionary trajectories. Central to our efforts is understanding the subtle roles of these adaptive markers. Our goal is to provide insights into their effects on host adaptation and virulence in AIVs and to translate these findings into a reliable quantitative risk assessment.

In assessing viral growth, we observed a strong correlation between growth speed (GS) values and traditional viral growth kinetics. The *R*^2^ values, ranging from 0.85 to 0.98, affirm the reliability of our non-linear regression model for such studies. This utility of the model extends to measuring and comparing replication capacities across different mutants. For example, while the PA_Q556R_ single mutant indicated a subdued GS, its combination with PB2_D701N_ displayed one of the stronger GS values, suggesting intricate interactions between mutations. Mathematical modelling allowed us to quantify GS values for each viral variant, establishing a framework for assessing viral growth dynamics. Intriguingly, while some mutants like double or triple combinations showed enhanced replication abilities as previously reported [44–47], the RG452 virus bearing the PB2_Q591K/D701N_ double mutations presented an anomaly with diminished replication efficiency when contrasted with its PB2_D701N_ single mutant counterpart. This unique observation, potentially highlighting an interplay between the two mutations influenced by PB1_P708S_, calls for deeper investigation.

The competitive selection kinetics studies provide an unprecedented quantitative comparison of individual and combined mammalian adaptive markers’ impact on host specificity. Furthermore, our calculated competitive selection rate (CSR) quantifies these effects, elucidating antagonistic, additive, and synergistic relationships between mutations. The finding that the RG452-PB2_Q591K/D701N_ double mutant registered CSR values similar to the single RG452-PB2_D701N_ mutant uncovers a potential cancellation effect. These complex interactions underscore the necessity for nuanced risk assessment and emphasize the ability of virus to adapt and evolve within the host. While our recombinant viruses were designed to limit mutation acquisition, additional mutations in the polymerase genes were observed during *in vitro* passages (Supplementary Table 8). Such mutations could potentially affect the growth and competitive properties of both the wild-type and mutant recombinant viruses, potentially impacting the precise competitive properties of the adaptive markers.

Understanding the roles and dynamics of three distinct types of influenza A viral RNAs (vRNA, cRNA, and mRNA) in the life cycle of the virus within mammalian cells is key to unravelling the infectious properties of virus. Building upon previous studies that analyzed the kinetic behaviour of these RNAs [48–52], our work delves deeper into their cellular functions, especially in the context of potential viral adaptations. Our statistical model effectively bridges experimental viral RNA expression data with quantitative values, facilitating straightforward comparisons of overall RNA replicative properties across viruses with adaptive markers. The model consistently captures the influence of various adaptive markers on RNA levels across all RNA types, underscoring its accuracy. Furthermore, the RNA weight model introduced in this study clarifies the impact of each RNA type on viral attributes. This provides insight into the interplay between RNA expression and viral dynamics, enhancing the precision of the RNA load value. The RG452-PA_Q556R_ mutation demonstrated a moderate impact on the GS and CSR but showed the highest RNA load value, particularly for vRNA, among the single mutants. This suggests an enhancing role in viral genome replication, potentially through interaction with the PB1 protein [22]. The interaction between PB2_Q591K_ and PB2_D701N_ mutations was notably antagonistic in RNA load value while the triple mutant (RG452-PB2_Q591K/D701N_ + PB1_P708S_) displayed significantly elevated RNA levels by adding the PB1_P708S_ mutation. This suggests the intricate interactions between these mutations are pivotal for viral adaptation, aligning with the observations of this triple mutant post-adaptation in a mammalian model [6, 20]. Such results underscore the distinctive impact specific mutations exert on RNA replication within the virus. The alignment between predictions of our model and the actual data validates its robustness, enriching our comprehension of the factors governing replication and competition in the influenza A virus.

We also developed a set of quantitative risk scores that serve as a valuable tool for evaluating how likely different viral strains are to adapt and cause outbreaks. The identification of a particular mutation, PB2_D701N_, as having the highest associated risk emphasizes its importance and suggests it could be a focal point for future surveillance and containment efforts. Validating these findings with another wild-type virus, RG468, further bolsters credibility of our model and demonstrates its broader applicability. Despite these advancements, our work leaves some questions unanswered. The utility of the model for other viral strains or host species remains to be explored, and further research could help adapt our insights to human contexts, potentially aiding in the development of more effective vaccines and antiviral therapies. Our research also acknowledges the inherent limitations posed by the nature of influenza viruses. While our study employed clonal recombinant viruses, we cannot rule out the acquisition of secondary mutations during replication in the host, which could potentially alter phenotypic outcomes. Recognizing this, we emphasize the need for future studies to delve into this area of viral evolution. Moreover, while the maH5N8 viruses used in our research belong to clade 2.3.4.4a [53] and lack evidence of human infection, their rapid pathogenic evolution in mammalian models necessitates further examination. This is particularly pressing in light of recent human infections caused by H5 viruses from clade 2.3.4.4b [54]. Our study’s insights provide a foundation for such investigations and highlight the importance of continuous research in tracking and understanding the dynamic nature of avian influenza viruses.

In conclusion, our study on the HPAI H5N8 virus provides critical insights into the dynamics of mammalian adaptive markers and their implications in viral pathogenesis and interspecies transmission. The comprehensive model we developed, integrating experimental data with mathematical analysis, not only enhances our understanding of H5N8 but also establishes a foundation for studying other rapidly evolving infectious agents. Our established quantitative risk scoring system offers a sophisticated approach to assessing the potential impact of specific genetic mutations on viral behaviour. This tool is particularly relevant in the current period, where rapid mutation and adaptation of viruses pose constant threats to global health. Our findings emphasize the need for ongoing monitoring and analysis of viral evolution, especially in the context of emerging zoonotic diseases. The methodologies and analytical frameworks we utilized can be adapted and applied to other infectious agents, providing a valuable repertoire for early detection, risk assessment, and strategic intervention planning. Such adaptability is vital for staying ahead of evolving pathogens and for developing timely, effective responses to emerging infectious disease threats. In a time characterized by frequent outbreaks of novel pathogens, our research adds to the collection of tools necessary for understanding and addressing these challenges. By offering a model that bridges traditional virological studies with advanced quantitative analysis, we aim to boost the predictive capabilities and preparedness of the global health community, thereby contributing to more effective strategies for managing and mitigating the impacts of infectious diseases.

## Ethical statement

All animal studies were carried out under the approval of the Institutional Animal Care and Use Committee of Chungbuk National University, Republic of Korea (CBNUA-1659-22-01).

## Supplementary Material

Supplementary_Revised-clean
