# Supplementary material for: Mammalian adaptation risk in HPAI H5N8: a comprehensive model bridging experimental data with mathematical insights
Source: Emerg Microbes Infect. 2024 Apr 4;13(1):2339949. doi: 10.1080/22221751.2024.2339949 (PMC11022924; doi:10.1080/22221751.2024.2339949)
Supplement: Supplementary_Revised-clean [file TEMI_A_2339949_SM5135.docx]

**Supplementary Table 1:** Compilation of single and combined adaptive markers for RG452 and RG468 generated using reverse genetics.

| Virus | PB2 | | | PB1 | PA | |
| --- | --- | --- | --- | --- | --- | --- |
|  | 591 *^a^* | 627 | 701 | 708 | 97 | 556 |
| RG452-WT | Q*^b^* | E | D | P | T | Q |
| RG452-PB2_D701N_ | - | - | N | - | - | - |
| RG452-PB2_Q591K_ | K | - | - | - | - | - |
| RG452-PB1_P708S_ | - | - | - | S | - | - |
| RG452-PA_Q556R_ | - | - | - | - | - | R |
| RG452-PB2_D701N/Q591K_ | K | - | N | - | - | - |
| RG452-PB2_D701N_ +PB1_P708S_ | - | - | N | - | - | R |
| RG452-PB2_D701N/Q591K_+PB1_P708S_ | K | - | N | S | - | - |
| RG468-WT | Q | E | D | P | T | Q |
| RG468-PB2_E627K_ | - | K | - | - | - | - |
| RG468-PB1_P708S_ | - | - | - | S | - | - |
| RG468-PA_T97I_ | - | - | - | - | I | - |
| RG468-PB2_E627K_+PB1_P708S_ | - | K | - | S | - | - |
| RG468-PB2_E627K_+PA_T97I_ | - | K | - | - | I | - |

*^a^* The number refers to the specific amino acid position within the indicated protein.

*^b^* The abbreviations represent specific amino acids.

PB2; polymerase basic protein 2, PB1; polymerase basic protein 1, PA; polymerase acidic protein

**Supplementary Table 2:** Primers for site-directed mutagenesis of RG452 and RG468 single and combined adaptive markers.

| Protein | Amino acid substitution | Primer name | Sequence (5' to 3') | Base pair |
| --- | --- | --- | --- | --- |
| PB2 | R*^a^*591*^b^*K | PB2 R591K F | GAGAGAGTGGTTGTA**A**GCATTGACCGTTTCTTGCG | 35 |
|  |  | PB2 R591K R | TACAACCACTCTCTCAGTGCTGGAA | 25 |
|  | E627K | PB2 E627K F | GAATGTAAGAGGCTC**A**GGAATGAGAATACTCGTAA | 35 |
|  |  | PB2 E627K R | AGCCTCTTACATTCACGGTCAGGGAA | 26 |
|  | D701N | PB2 D701N F | ATTCTGGGCAAGGAG**G**ACAGAAGATATGGACCAGC | 35 |
|  |  | PB2 D701N R | CTCCTTGCCCAGAATTAGGAACCCCCTCA | 29 |
| PB1 | P708S | PB1 P708S F | TAGCTCATACAGGAGG**C**CAGTTGGAATCTCCAGTA | 35 |
|  |  | PB1 P708S R | CTCCTGTATGAGCTACTAGGGAAGAA | 26 |
| PA | T97I | PA T97I F | AAGGGAGAGACCGAACGAT**A**GCTTGGACAGTGGTA | 35 |
|  |  | PA T97I R | TTCGGTCTCTCCCTTCAATTATCTC | 25 |
|  | Q556R | PA Q556R F | AACTGCAATAGGCC**G**AGTATCAAGACCCATGTTTC | 35 |
|  |  | PA Q556R R | GGCCTATTGCAGTTCGTAGGAGCATGTCT | 29 |

*^a^* The abbreviations represent specific amino acids.

*^b^* The number refers to the specific amino acid position within the indicated protein.

The underscore '_' denotes the nucleotide mutation introduced to achieve a specific amino acid substitution.

PB2; polymerase basic protein 2, PB1; polymerase basic protein 1, PA; polymerase acidic protein

**Supplementary Table 3:** Gene-specific primers employed for cDNA synthesis and real-time qPCR analysis of the viral NP RNA segment.

| **Target gene (NP)** | | **Primer name** | **Sequence (5' to 3')** |
| --- | --- | --- | --- |
| vRNA | Reverse Transcription | vRNA 730F | GGCCGTCATGGTGGCGAATCAAGTGCGAGAAAGCAGGAA |
|  | Real-time PCR | vRNA tag | GGCCGTCATGGTGGCGAAT |
|  |  | vRNA 730R | AACACAAGCAGGCAGACAAG |
| cRNA | Reverse Transcription | cRNA 1565R | GCTAGCTTCAGCTAGGCATCAGTAGAAACAAGGGTATTTTTCTT |
|  | Real-time PCR | cRNA tag | GCTAGCTTCAGCTAGGCATC |
|  |  | cRNA 1375F | AAAGGCAACGAACCCGAT |
| mRNA | Reverse Transcription | mRNA dTR | CCAGATCGTTCGAGTCGTTTTTTTTTTTTTTTTTTACTCCAATTC |
|  | Real-time PCR | mRNA tag | CCAGATCGTTCGAGTCGT |
|  |  | mRNA 927F | TCTATGAGGGAAGAGTACCGGCAG |

**Supplementary Table 4:** Growth speed and R-Squared values for individual and combined adaptive markers.

| **Virus** | **Growth Speed** | **R-Squared** |
| --- | --- | --- |
| RG452-WT | 0.88 | 0.8507 |
| RG452-PB2_R591K_ | 1.49 | 0.9599 |
| RG452-PB2_D701N_ | 2.34 | 0.9818 |
| RG452-PB1_P708S_ | 1.53 | 0.9731 |
| RG452-PA_Q556R_ | 1.33 | 0.9490 |
| RG452-PB2_D701N/R591K_ | 2.18 | 0.9002 |
| RG452-PB2_D701N_+PA_Q556R_ | 2.56 | 0.9694 |
| RG452-PB2_D701N/R591K_+PB1_P708S_ | 2.55 | 0.9832 |

**Supplementary Table 5:** Kinetics and rates of competitive selection for single and combined adaptive markers in RG452, evaluated at dilutions of 1:100, 1: 1,000, and 1: 10,000 relatives to the wild type and RG viruses.

| **Virus** | **1:100** | | **1:1,000** | | **1:10,000** | | **CSR***^b^* **(AUC)** |
| --- | --- | --- | --- | --- | --- | --- | --- |
|  | **CSK***^a^* | **R-Squared** | **CSK** | **R-Squared** | **CSK** | **R-Squared** |  |
|  |  |  |  |  |  |  |  |
| RG452-PB2_R591K_ | 60.40 | 0.8842 | 0.00 | 0.0000 | 0.00 | 0.0000 | 30.20 |
|  |  |  |  |  |  |  |  |
| RG452-PB2_D701N_ | 128.55 | 0.9170 | 94.62 | 0.9102 | 33.15 | 0.7074 | 178.71 |
|  |  |  |  |  |  |  |  |
| RG452-PB1_P708S_ | 98.14 | 0.9588 | 55.68 | 0.7474 | 10.39 | 0.4286 | 110.03 |
|  |  |  |  |  |  |  |  |
| RG452-PA_Q556R_ | 115.09 | 0.9105 | 47.66 | 0.9220 | 0.00 | 0.0000 | 105.13 |
|  |  |  |  |  |  |  |  |
| RG452-PB2_D701N/R591K_ | 125.36 | 0.8478 | 92.20 | 0.9202 | 52.21 | 0.9124 | 179.32 |
|  |  |  |  |  |  |  |  |
| RG452-PB2_D701N_+PA_Q556R_ | 289.36 | 0.9932 | 195.32 | 0.9435 | 55.96 | 0.9013 | 375.61 |
|  |  |  |  |  |  |  |  |
| RG452-PB2_D701N/Q591K_+PB1_P708S_ | 340.37 | 0.9915 | 286.46 | 0.9930 | 113.27 | 0.9467 | 514.71 |
|  |  |  |  |  |  |  |  |

*^a^* Values were derived using nonlinear regression based on the proportion of the competitively selected marker observed at specific dilution points.

*^b^* Data represents the Area Under Curve (AUC) for CSK values across dilutions of 1:100, 1: 1,000, and 1: 10,000.

CSK; competitive selection kinetics, CSR; competitive selection rate

**Supplementary Table 6:** Normalized metrics to 1 for growth speed, competitive selection rate, and RNA load values for individual and combined adaptive markers in RG452 viruses.

| **Virus** | **Growth Speed** | **Competitive Selection Rate** | **RNA load** |
| --- | --- | --- | --- |
| RG452-WT | 0.0000 | 0.0000 | 0.0000 |
| RG452-PB2_Q591K_ | 0.3640 | 0.0585 | 0.0000 |
| RG452-PB2_D701N_ | 0.8681 | 0.3377 | 0.2558 |
| RG452-PB1_P708S_ | 0.3830 | 0.2128 | 0.1257 |
| RG452-PA_Q556R_ | 0.2635 | 0.2038 | 0.2532 |
| RG452-PB2_D701N/Q591K_ | 0.7698 | 0.3437 | 0.0596 |
| RG452-PB2_D701N_+PA_Q556R_ | 1.0000 | 0.7312 | 0.3166 |
| RG452-PB2_D701N/Q591K_+PB1_P708S_ | 0.9896 | 1.0000 | 1.0000 |

**Supplementary Table 7:** Normalized metrics to 1 for growth speed, competitive selection rate, and RNA load values for individual and combined adaptive markers in RG468 viruses.

| **Virus** | **Growth Speed** | **Competitive**  **Selection Rate** | **RNA load** |
| --- | --- | --- | --- |
| RG468-WT | 0.0000 | 0.0000 | 0.0000 |
| RG468-PB2_E627K_ | 0.9869 | 0.7891 | 0.3491 |
| RG468-PB1_P708S_ | 0.5197 | 0.1625 | 0.1320 |
| RG468-PA_T97I_ | 0.2964 | 0.1631 | 0.1712 |
| RG468-PB2_E627K_+PB1_P708S_ | 0.9470 | 1.0000 | 0.5528 |
| RG468-PB2_E627K_+PA_T97I_ | 1.0000 | 0.8196 | 1.0000 |

**Supplementary Table 8:** Substitutions Detected in Recombinant Viral Polymerase Genes During In Vitro Passaging.

| Virus | Mutations in PA  (%) | Mutations in PB1  (%) | Mutations in PB2  (%) |
| --- | --- | --- | --- |
| RG452-PB2_R591K_ | - | - | N540I (28.5) |
| RG452-PB2_D701N_ | - | - | R597G (27.6) |
| RG452-PB1_P708S_ | - | - | - |
| RG452-PA_Q556R_ | - | - | - |
| RG452-PB2_D701N/R591K_ | - | - | P515L (21.5)  N540I (16.9 – 22.8)  T745I (34.7 – 39.5) |
| RG452-PB2_D701N_+PA_Q556R_ | - | - | - |
| RG452-PB2_D701N/R591K_+PB1_P708S_ | - | - | T745I (23.3 – 37.6) |

Amino acid mutations in polymerase complex genes (PB2, PB1, and PA) of recombinant influenza viruses, with a detection threshold set at 3% during deep sequencing.


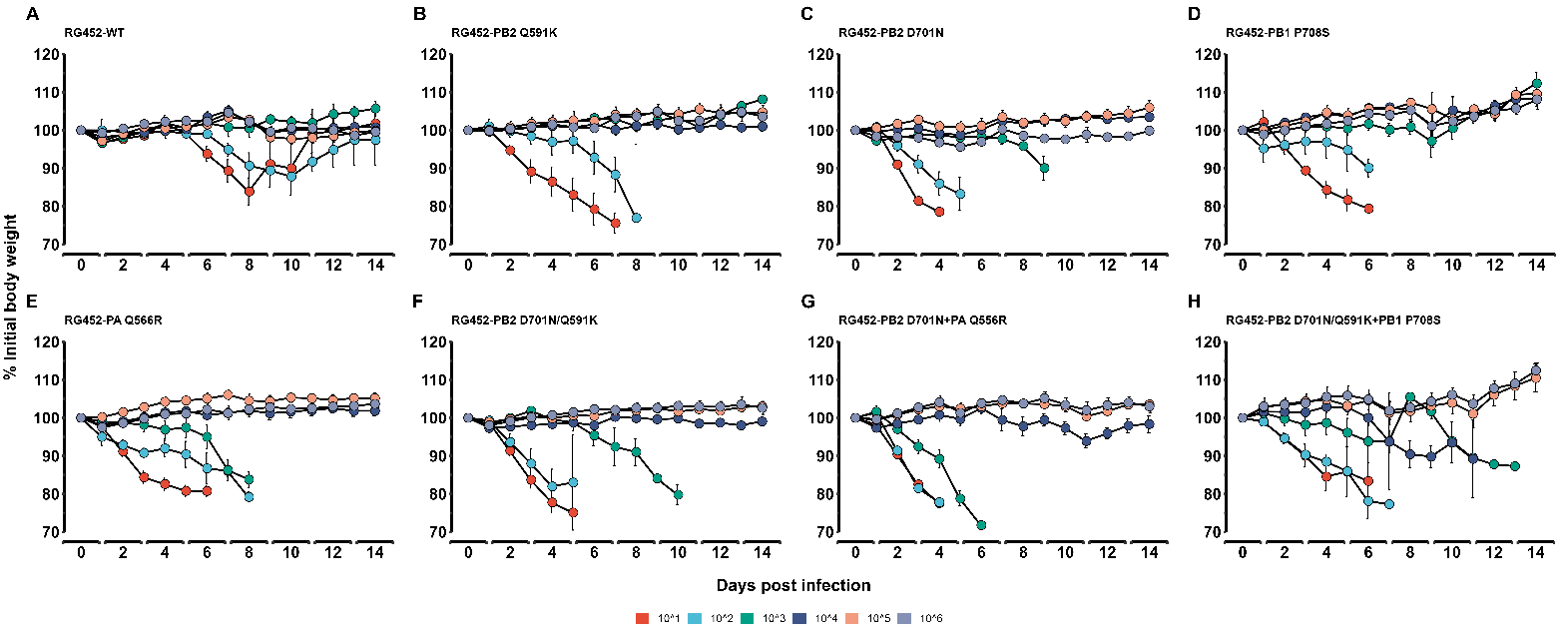


**Fig. S1:** Monitoring of Body Weight Fluctuations in BALB/c Mice Post-Infection with RG452 Variants. (A-H) Weight Change Dynamics Post-RG452 Infection: The sequential panels display the variation in body weight of BALB/c mice after they were administered with dilutions ranging from 10^-6 to 10^-1 of the RG452 recombinant viruses, each possessing distinct adaptive markers: (A) RG452-WT, (B) RG452-PB2_Q591K_, (C) RG452-PB2_D701N_, (D) RG452-PB1_P708S_, (E) RG452-PA_Q556R_, (F) RG452-PB2_D701N/Q591K_, (G) RG452-PB2_D701N+PAQ556R_, (H) RG452-PB2_D701N/Q591K+PB1P708S_. For a span of 14 days post-infection (DPI), alterations in body weight were meticulously observed and represented as a percentage (%) relative to the starting body weight.

**
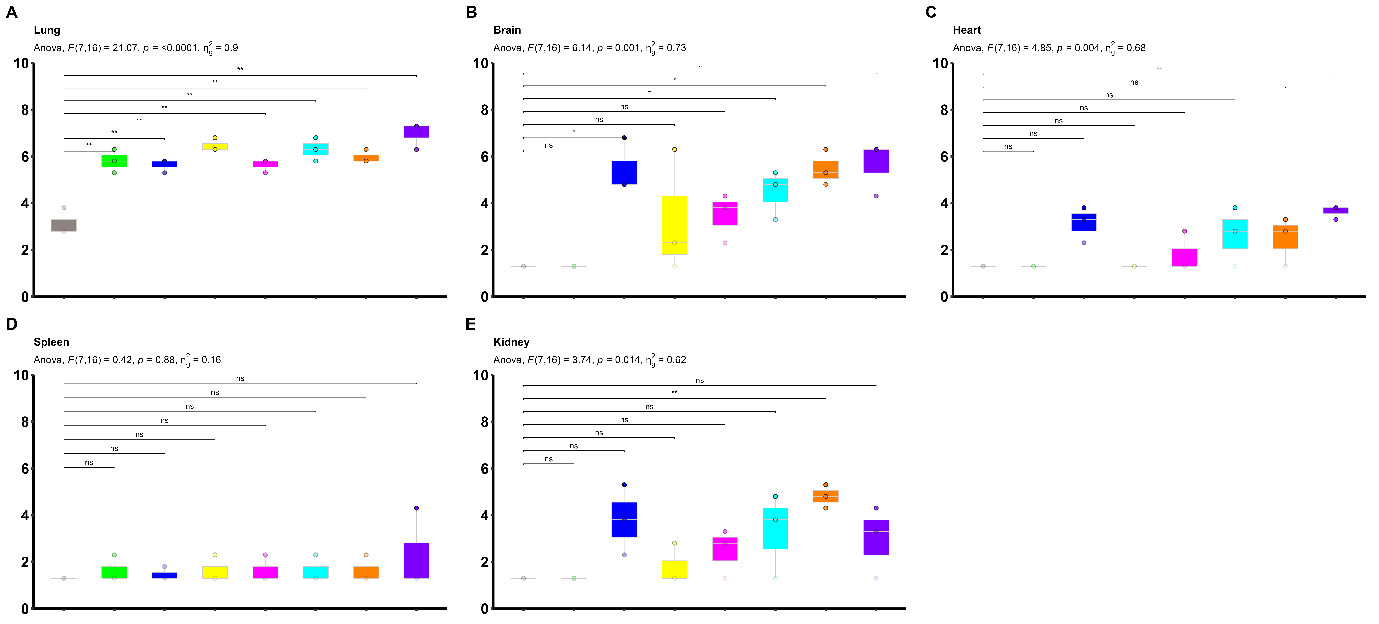
**

**Fig. S2:** Analysis of Viral Dissemination in Organ Tissues of BALB/c Mice Infected with RG Variants. Post-infection with single and combinational adaptive variants of the RG virus in BALB/c mice, viral titers in various organs were ascertained at 5 days post-infection (DPI). Specifically, the viral loads in: (A) Lung, (B) Brain, (C) Heart, (D) Spleen, (E) Kidney are showcased. The determined viral titers are represented as log_10_ PFU/mL. The significance of findings is marked by: ns (not significant), *p < 0.05, **p < 0.01.
